# Computational Analysis of the Soluble Form of the Intracellular Chloride Ion Channel Protein CLIC1

**DOI:** 10.1155/2013/170586

**Published:** 2013-09-08

**Authors:** Peter M. Jones, Paul M. G. Curmi, Stella M. Valenzuela, Anthony M. George

**Affiliations:** ^1^School of Medical and Molecular Biosciences, University of Technology Sydney, P.O. Box 123, Broadway, NSW 2007, Australia; ^2^School of Physics, University of New South Wales, Sydney, NSW 2052, Australia; ^3^St Vincent's Centre for Applied Medical Research, St Vincent's Hospital, Darlinghurst, NSW 2010, Australia

## Abstract

The chloride intracellular channel (CLIC) family of proteins has the remarkable property of maintaining both a soluble form and an integral membrane form acting as an ion channel. The soluble form is structurally related to the glutathione-S-transferase family, and CLIC can covalently bind glutathione via an active site cysteine. We report approximately 0.6 *μ*s of molecular dynamics simulations, encompassing the three possible ligand-bound states of CLIC1, using the structure of GSH-bound human CLIC1. Noncovalently bound GSH was rapidly released from the protein, whereas the covalently ligand-bound protein remained close to the starting structure over 0.25 *μ*s of simulation. In the unliganded state, conformational changes in the vicinity of the glutathione-binding site resulted in reduced reactivity of the active site thiol. Elastic network analysis indicated that the changes in the unliganded state are intrinsic to the protein architecture and likely represent functional transitions. Overall, our results are consistent with a model of CLIC function in which covalent binding of glutathione does not occur spontaneously but requires interaction with another protein to stabilise the GSH binding site and/or transfer of the ligand. The results do not indicate how CLIC1 undergoes a radical conformational change to form a transmembrane chloride channel but further elucidate the mechanism by which CLICs are redox controlled.

## 1. Introduction

The chloride intracellular channel (CLIC) family of proteins consists of six distinct members in vertebrates: CLIC1-6. Most CLICs are localized to intracellular membranes and are known to participate in protein-protein interactions, particularly with cytoskeletal components [1–5]. The oxidation state of CLIC has been found to affect the activity of associated chloride channels, and CLICs have been shown *in vitro* to insert from the aqueous phase into phospholipid membranes, where they can function as an anion channel [6, 7]. CLIC proteins can also act as substrates of various kinases [8], and, as has been shown for CLIC5B, tyrosine phosphorylation may affect activity [9, 10]. Although these proteins have been linked to functions including apoptosis and pH and cell cycle regulation [11–13], their exact role in normal cell physiology is uncertain.

**Figure 1:**
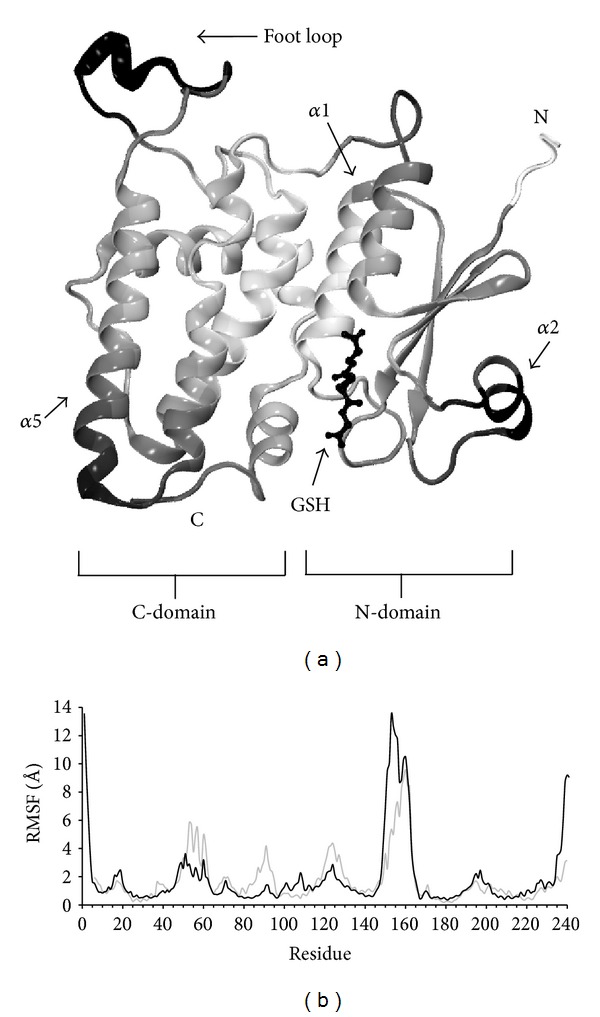
Structure and dynamics of human CLIC1. (a) Ribbon representation of the human CLIC1 structure used in the simulations. GSH is shown in stick form. The foot loop (residues 147–164) was modeled using coordinates from nonliganded CLIC1 (PDB 1K0 M). Secondary structural elements discussed in the text are as indicated. Residues are shaded according to the combined fluctuations for slow modes 1–5 from the GNM analysis with white, low and black, high fluctuations. (b) Per residue plot of average of RMS fluctuations of C*α* atoms from all simulations (black). The fluctuation profile predicted by GNM analysis for slow modes 1–5 is in grey. The GNM data are scaled linearly to fit the range of the MD data for the purposes of comparison.

The CLIC family is defined by a C-terminal core segment of approximately 230 amino acids that is highly conserved among all family members. Sequences upstream this core vary considerably among the various family members in length and sequence. CLIC1 has been most intensely studied [6, 14–17] and has only a few amino acids upstream of the conserved core that defines the CLIC family. CLIC1 has been shown to assume both soluble and integral membrane forms, which presumably differ radically in conformation. The crystal structure of a soluble monomeric form of CLIC1 was found to be a structural homologue of the glutathione-S-Transferase (GST) superfamily of proteins [6]. This soluble form of CLIC1 consists of two domains: the N-domain possessing a thioredoxin fold closely resembling glutaredoxin and an all *α*-helical C-domain, which is typical of the GST superfamily (Figure 1). CLIC1 contains an intact glutathione-binding site that was shown to covalently bind glutathione (GSH) via a conserved CLIC cysteine residue, Cys24. This led to the suggestion that CLICs are likely to be GSH-dependent redox-active proteins. The structure of the soluble form of CLIC1 indicates that it closely resembles that of the Omega class GST [18]. The Omega class GST GSTO1-1 modulates ryanodine receptors, which are the calcium release channels in skeletal and cardiac sarcoplasmic reticulum [19].

In order to investigate the role and molecular mechanism of CLIC1, we performed molecular dynamics (MD) simulations of the three possible ligand-bound states of the molecule: no ligand (apo); GSH noncovalently bound; and GSH covalently bound. The simulations revealed that CLIC1 undergoes distinct structural changes in each of the different states that, together with experimental data, support a model for CLIC function in which GSH is transferred to the protein by an unknown enzyme.

## 2. Experimental Procedures

The crystal structure of soluble monomeric GSH-bound CLIC1 (PDB 1K0N, monomer A) was used as the starting structure for all simulations. Residues 153 to 162 are disordered in this structure and the coordinates for this region were generated using the apo structure (PDB 1K0M). The GSH-bound CLIC structure (1K0N) was determined by soaking crystals from which the apo structure (1K0M) was derived with oxidised glutathione; glutathione binding induced only small changes in the protein structure (RSMD of 0.29 Å between monomer A in apo and glutathione bound). Engineered mutation Glu151Gly was reversed. Molecular Dynamics (MD) simulations were performed using the parallel MD program NAMD 2.6 [20] with the CHARMM27 force field [21] including *φ*/*ψ* cross-term map corrections [22] and the TIP3P model for water [23]. Parameters for GSH were derived from CHARMM protein parameters as described (http://www.ks.uiuc.edu/Research/namd/). The SHAKE algorithm was used to constrain the bonds containing hydrogens to their equilibrium length [24]. A cutoff of 12 Å (switching function starting at 10 Å) for van der Waals interactions and real space electrostatic interactions was used. Periodic boundary conditions were used and minimum distances between periodic images of the protein were 20 Å in all cases. The particle-mesh Ewald method [25] was used to compute long-range electrostatic forces with a grid density of approximately 1/Å^3^. An integration time step of 1.5 fs was used, permitting a multiple time-stepping algorithm to be employed in which interactions involving covalent bonds and short-range nonbonded interactions were computed every time step, whereas long-range electrostatic forces were computed every two time steps. Langevin dynamics were utilized to keep a constant temperature of 310 K in all simulations with a friction coefficient of 5 ps^−1^ on all nonhydrogen atoms. Constant pressure simulations at 1 atm were conducted using a Langevin piston method with a decay period of 100 fs and a damping timescale of 50 fs. An initial set of three simulations was performed with a 0.2 M NaCl solution and a second set of three equivalent simulations used minimum sodium ions to electrically neutralise the system.

The solvated starting structure was minimized using conjugate gradient minimization to a 0.5 kcal/mol·Å RMS gradient with all protein heavy atoms fixed. Water molecules and protein hydrogens were then further minimized during a 50 ps molecular dynamics run at 310 K, in which all protein heavy atoms were again fixed. This starting model was then minimized with harmonic positional constraints on the NC*α*CO backbone. A 100 kcal/mol·Å^2^ force constant was used to minimise the system to a 0.5 kcal/mol·Å RMS gradient. The constraints were gradually removed by subsequent minimizations to a 0.1 kcal/mol·Å rms gradient, scaling the initial force constants by factors of 0.5, 0.15, 0.05, and 0. The minimized structure was then heated from 50 K to 310 K in steps of 25 K using velocity reassignment during a 30 ps MD run. This equilibrated structure was used for the production run in which no restraints were applied.

Using the 1K0N monomer A structure, three simulations of the possible ligand bound states of the molecule were performed: (i) no ligand (apo1, 95 M steps = 142.5 ns); (ii) GSH covalently bound (cov1, 75 M steps = 112.5 ns); (iii) GSH noncovalently bound (ncv1, 48.7 M steps = 73 ns). A second set of simulations was performed in which the C-terminal residues 234–241 were modeled in an *α*-helical structure, such that these residues extended the C-terminal *α*-helix (residues 226–233). Coordinates for residues 234–241 were generated by structurally aligning a regular *α*-helix of suitable length with the C*α* atoms of residues 226–233 and then generating the side chains of residues 234–241 using the mutate function in Swiss-PdbViewer [26]. Using this starting structure, three simulations of the possible ligand bound states of the molecule were performed: (i) no ligand (apo2, 75 M steps = 112.5 ns); (ii) GSH covalently bound (cov2, 95 M steps = 142.5 ns); (iii) GSH noncovalently bound (ncv2, 10 ns). In addition, a third simulation with GSH noncovalently bound, using the crystal structure of human CLIC1 with the Q63E mutation reversed, was also performed (ncv3, 23 ns). The noncovalently ligand-bound simulations were terminated following release of the ligand. All simulations remained stable to completion. Coordinates were saved every 1000 integration steps (1.5 ps).

Principal component analysis (PCA) of the C*α* atom coordinate trajectory was used to identify and characterise global conformational transitions. PCA defines a set of eigenvectors (EVs) derived from the matrix of pairwise correlated motion of atoms. EVs are ranked according to the amplitude of the protein motions they describe, and in general, the first 1 to 4 EVs account for the most concerted 50% or more of protein fluctuations. PCA of the simulation *α*-carbon atom trajectories was performed using the GROMACS package [27]. N-terminal residues 1–5 and foot loop residues 145–165 were not included in the PCA analysis. Trajectory frames were first aligned to the starting structure using coordinates of the structural core (residues 25–37, 76–88, 93–98, 139–143, 167–186, and 205–215). Fluctuations were referenced to the average structure of the trajectory. Secondary structure of simulation trajectory frames was analysed using the method of Kabsch and Sander [28] as implemented in Simulaid (http://atlas.physbio.mssm.edu/~mezei/).

Calculation of the pKa of residue Cys24 was performed with the MCCE package [29, 30] using the single conformer protocol supplied with the program. Elastic network analyses were performed on the CLIC1 structure using the online facility [31] available at http://ignm.ccbb.pitt.edu/, using default settings. Calculation of electrostatic potential was performed using Delphi v4 [32]; electrostatic potential is expressed in units of kT/e (*k* = 0.001986577 kcal/mol·K). Sequence variability of CLICs, expressed as the Shannon entropy [33, 34], was calculated using the online Sequence Variability Server available at http://bio.dfci.harvard.edu/Tools/sva.html. A CLUSTALW alignment of the closest 100 sequences to human CLIC1, as determined from a BLAST search of the Swiss-Prot database, was used as input to the Sequence Variability Server. The variability in each column of the multiple sequence alignment is calculated using the Shannon entropy (*H*) function. All structural figures were prepared using VMD [35, 36], (available at http://www.ks.uiuc.edu/Research/vmd/) except for Figures 3(b), 3(c), and Figure 5(a), which were prepared using PyMol [37].

## 3. Results

In this study, we performed MD simulations of the three possible ligand-bound states of human CLIC1: (i) no ligand (apo); (ii) GSH noncovalently bound (ncv); and (iii) GSH covalently bound (cov). Two sets of simulations of these three states were performed: the first set using the crystal structure of human CLIC1 as the starting structure and the second set using the same structure, but with the C-terminal residues 234–241 modeled in an *α*-helical conformation (see below). In addition, a third simulation with GSH noncovalently bound, using the crystal structure of human CLIC1 with ligand-binding residue Glu63 changed to a glutamine, was also performed. The combined total of real-time simulated was approximately 0.6 *μ*s.

As a point of comparison for the MD analysis, Elastic Network (EN) analysis of the CLIC1 structure was also performed. The EN approach is a normal mode analysis based on a coarse-grained model in which the protein is modeled as a set of points, corresponding to the C*α* atom positions, connected to their nearest neighbours by simple harmonic springs [38]. This analysis can identify the principal correlated motions in proteins, which play a predominant role in effectuating functional motions. These are usually described within the 5 slowest modes [39], although not all EN modes necessarily represent functional motions. Based solely on the protein contact topology, EN analysis provides an independent methodology with which one can compare the results of the MD studies and can also provide insights into conformational transitions that occur on timescales beyond the reach of the atomistic simulations. Here, we employ both the Gaussian Network Model [40] and the Anisotropic Network Model (ANM; [41]) and discuss EN modes that correlate with the results of MD simulations. We also employ principal component analysis (PCA; see Methods) to analyse the simulation trajectories. PCA filters the most concerted global protein changes from random fluctuations and can be used to delineate and characterise motions linked to biological function.

### 3.1. MD and GNM Identify Dynamic Regions of the CLIC1 Structure

Figure 1(b) shows plots of the average-per-residue fluctuations from all the MD simulations and for comparison, the combined fluctuations from slow modes 1–5 from the GNM analysis. To give a structural reference, the CLIC1 structure in Figure 1(a) is shaded according to the value of the GNM data plotted. In general, the GNM and MD analyses are in agreement regarding the regions of the protein that undergo elevated fluctuations. In particular, the “foot loop” region (residues 146–165) is notable for its high mobility, consistent with its disordered nature in the crystal structure. The other regions with high fluctuations in the MD simulations are *α*-helix 2 and the loops at its N and C termini (residues 48–63) and the region of the loop joining *α*-helices 4b and 5. Perhaps the most notable difference between the two analyses is that GNM predicts high fluctuations for the loop at the C-terminus of *α*-helix 3 (residues 89–94, loop at top centre right in Figure 1(a)), in agreement with high temperature factors for this region in the crystal structure, although in general, only moderately elevated fluctuations were observed for this region in the MD simulations. We note that in the 1.4 Å crystal structure of apo CLIC1, there are two monomers in the asymmetric unit with Pro91 adopting a cis conformer in monomer A, whereas Pro91 in monomer B appears to be in both *cis* and *trans* conformations [6]. 

### 3.2. Conformational Changes in the Apo State

**Figure 2:**
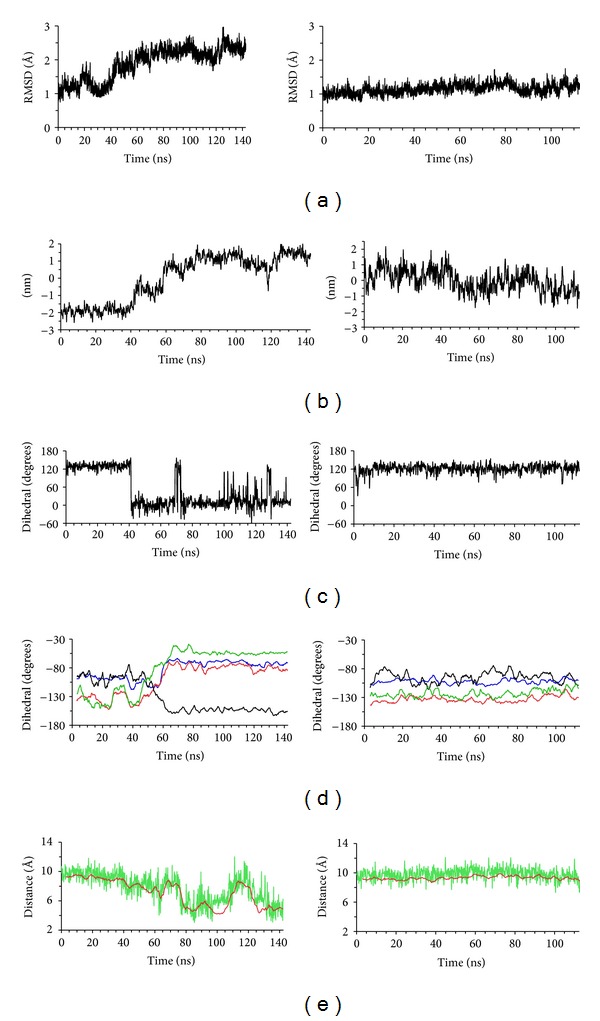
Time course of conformational changes in the apo and covalently ligand-bound simulations. Data from the apo1 simulation are in the left panels and those from the cov1 simulation are in the right panels. All measurements were taken at 150 ps intervals. (a) RMS deviation of C*α* atoms from the starting structure (not including residues 1–5 or 146–165). (b) Projection of PCA EV1 from the C*α* trajectory (not including residues 145–165). (c) Backbone *φ* dihedral angle of residue Asn23. (d) Backbone *ψ* dihedral angles of residues 194 (blue), 195 (red), 197 (black), and 198 (green). (e) Distance between the hydroxyl oxygen of Tyr233 and the sulfur atom of Cys24 (green) and moving average over 20 150 ps intervals of distance between the hydroxyl oxygen of Tyr233 and the side chain *β*-carbon atom of Cys24 (red).

Two initial simulations of 75 M time-steps (112.5 ns) of the apo (apo1) and covalently bound ligand state (cov1) were performed. The apo1 simulation was continued for a further 20 M timesteps (to 142.5 ns), as discussed below.  Figure 2 shows results from the apo1 and cov1 simulations, which illustrate and characterise differences between them. A significant conformational change occurred in the apo state that did not occur in the covalently bound ligand state, the latter remaining closer to the starting structure throughout the simulation. This is illustrated in Figure 2(a), which show the time course of the rms deviation of the C*α* coordinates relative to the starting structure.  Figure 2(b) shows the time course of the projection of PCA EV1, which characterises the most concerted global changes in the protein. This measure also shows clearly that for the apo1 simulation EV1 describes an equilibration to a significantly different conformation from the starting structure, whilst in contrast concerted global change in the ligand-bound protein is not as marked.

**Figure 3:**
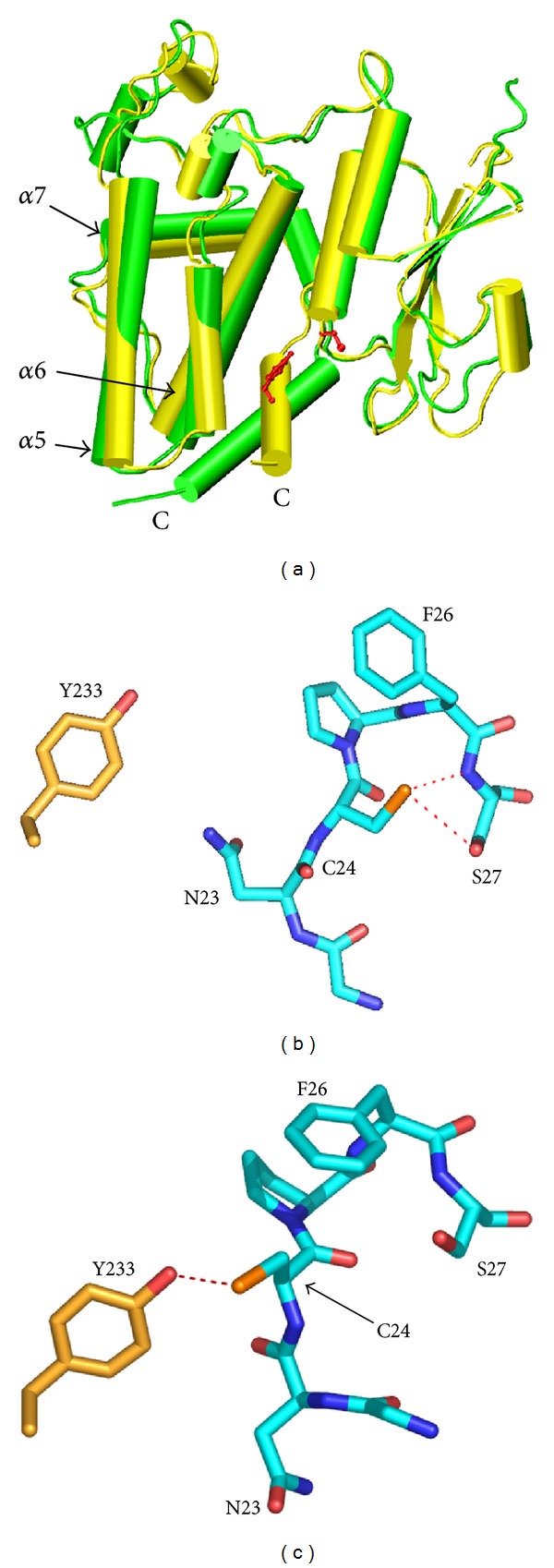
Conformational changes around the GSH-binding site in the apo1 simulation. (a) Global structural changes. Stereo pair in cartoon representation of the superimposed trajectory frames corresponding to the maximum (yellow, *t* = 7.6 ns) and minimum (green, *t* = 129.4 ns) projection structures for EV1 from the apo1 simulation. The view is similar to that in Figure 1. Tyr233 and Cys24 from the maximum projection structure are depicted in ball and stick and coloured red. Secondary structural elements discussed in the text are as indicated. (b)-(c) Interaction of Tyr233 and Cys24. Residues shown in stick form with carbon coloured cyan, oxygen red, nitrogen blue, and sulphur orange. Hydrogen bonds are shown as red dashed lines. (b) *t* = 10 ns, (c) *t* = 142.5 ns.

The large conformational change in the apo simulation involved concerted alterations in several areas of the protein. The most notable of these involved an alteration in the orientation of the C-terminal *α*-helix 9 (residues 226–233), which pivoted about its N-terminus. In addition, the C-terminal tail formed *α*-helical structure, extending *α*-helix 9 (Figure 3(a)), resulting in an *α*-helix between residues 226–240; this *α*-helix formed a hydrophobic face oriented toward the GSH binding site. The loop region joining *α*-helices 6 and 7 (residues 195–198) adopted a more compact conformation by way of alterations in backbone dihedral angles (Figure 2(d)). This allowed the C-terminus of *α*-helix 6 to move away from the active site, thereby in turn facilitating the alterations in the orientation of the C-terminal helix (Figure 3(a)).

The conformational changes in the apo simulation resulted in the direct interaction between the hydroxyl group of Tyr233 and the conserved active site Cys24 (Figures 2, 3(b), and 3(c)). The interaction between Tyr233 and Cys24 was also facilitated by an alteration in the conformation of residue Asn23 at the N-terminus of *α*-helix 1 (Figure 2(c)), such that its side chain moved away from the open cavity around the active site, enabling the side chain of Cys24 to point toward Tyr233 (Figure 3(c)). At the end of the initial apo simulation (*t* = 112.5 ns), the distance between Tyr233 and Cys24 was increasing, and so another 20 M steps of simulation were performed to ensure that the structure was indeed equilibrating toward a stable interaction of Tyr233 and Cys24 (Figure 2).

The conformational changes in the C-terminal *α*-helix 9 in the apo simulation, that resulted in the direct interaction of Tyr233 and Cys24, appeared connected to the disengagement of the C-terminal tail (residues 237–241) from a number of electrostatic and hydrophobic contacts with residues vicinal to the C-terminus of *α*-helix 6 (Figure 3(a)). Although this change from the crystal structure did not occur in the covalently ligand-bound simulation, it appeared possible that had the C-terminal tail similarly disengaged, the conformational changes observed in the apo simulation might also have occurred in the covalently ligand-bound CLIC. In other words, because of the stochastic nature of protein dynamic processes, it is possible that the covalently ligand-bound simulation did not have sufficient time to undergo the conformational change observed in the apo state. 

In order to find further evidence that the observed differences between the apo and covalently ligand bound simulations were due to their ligand-bound state, a second set of simulations of 75 M timesteps was performed in which C-terminal residues 226–241 were modeled as a continuous *α*-helix in the starting structure. As a further test of the robustness of the results, the second set of simulations used only the minimum counterions required to electrically neutralise the system rather than a 0.2 M electrolyte solution. As illustrated in Supplementary Figure 1(a), the interaction of Tyr233 and Cys24 and the concomitant protein conformational changes observed in the initial apo simulation were essentially repeated in the second apo simulation, however, as in the first simulations, did not occur in the second covalently ligand-bound simulation. The cov2 simulation was extended for another 20 M time steps to ensure that it had equilibrated. Notably, in the apo2 simulation, the initial *α*-helical structure imposed on the C-terminal residues was disrupted during the early part of the simulation, but reformed consistently in the second half of the simulation (Supplementary Figure 1(b)).

**Figure 4:**
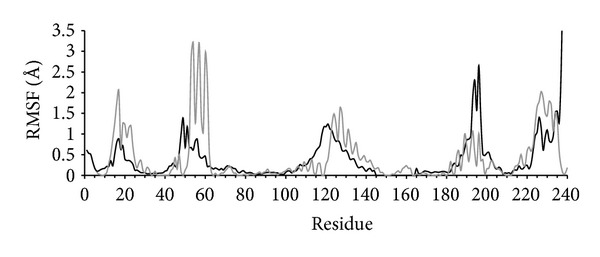
Correspondence between MD and GNM analysis of protein dynamics. Per residue plot of RMS fluctuations of C*α* atoms derived from PCA of apo simulation 1 (EV1) are in black. Foot loop residues 145–165 were not included in the PCA analysis. The fluctuation profile predicted by GNM analysis for slow mode 5 is in grey. The GNM data are scaled to fit the range of the MD data for the purposes of comparison.

Gaussian network model (GNM) analysis of the CLIC1 structure provides additional support for the idea that the conformational changes observed in the apo simulations describe functional transitions intrinsic to the protein.  Figure 4 illustrates how residues involved in motions described by slow mode 5 from the GNM analysis generally correspond with those derived from the PCA describing conformational changes in the apo1 simulation (EV1), as discussed above. Although the correspondence is not exact with respect to the regions involved in the transitions, it is notable that GNM EV5 describes correlated motions (same direction) of the C-terminal *α*-helix 9 and *α*-helix 7, anticorrelated with motions of the region of the N-terminal of *α*-helix 5 and *α*-helix 2, on the opposite side of the mouth around the active site (Supplementary Figure 2). This is similar to the transition that occurs during the equilibration in the apo simulations (Figure 3(a)). Supplementary Figure 3 further illustrates how the first 1–5 GNM modes contribute dominantly to the most concerted fluctuations observed in the apo1 and cov1 simulations.

### 3.3. Conformational Changes in the Unliganded State Result in Reduced Reactivity of the Active Site Thiol

We examined the effect of the change in proximity of Tyr233 and Cys24 on the pKa of Cys24 using continuum electrostatic calculations. The pKa of Cys24 was calculated in the 10 ns equilibrated structure and the final structure (*t* = 142.5 ns) from the apo1 simulation. The calculated pKa of Cys24 in the 10 ns equilibrated structure was 7.0 and in the final structure 8.54. This indicates that the pKa of Cys24 is shifted significantly toward neutrality in both structures, consistent with its role in forming a disulphide bond with GSH [42]. It also indicates that the active site thiol will more readily lose its proton in the 10 ns equilibrated structure than the final structure, following the global conformational change described above.

The breakdown of the calculated energy terms that contributes to the pKa of Cys24 indicated that the loss of interactions of the Cys24 side chain with the peptide backbone and with Ser27, in the latter part of the apo simulation (Figure 3(c)), accounts for the change in pKa between the 10 ns equilibrated structure and the final structure (*t* = 142.5 ns) from apo simulation 1 (Supplementary Table 1). This in turn is due to the conformational changes in both the peptide backbone of residues 23-24 and the sidechain rotamer of Cys24, which occur during the apo simulation (Figures 2, 3(b), and 3(c)). It is notable that the disposition of the Cys24 side chain in the 10 ns equilibrated apo structure is essentially the same, with respect to atoms of Ser27 and the electrostatic dipole of *α*-helix 1, as observed in the GSH-bound crystal structure. Thus, the data suggest that the Cys24 thiol would be less reactive in the conformation observed in the final apo structure, where it interacts with Tyr233, than in the conformation it is in when GSH is covalently bound.

The data also highlight the likely role of Ser27 in activating the active site thiol. In addition to these changes in the vicinity of the active site, it is notable that in both apo simulations, Phe26, which is part of the conserved Cys-Pro-(Phe/Tyr)-(Cys/Ser) GSH-binding motif found in the glutaredoxins [43], rotates into the area occupied by the ligand when it is covalently bound. This occurs transiently in the latter half of the apo simulation 2 but occurs in a more sustained manner in apo simulation 1 (Supplementary Figure 4).

Finally, the CLIC1 sequence was analysed for possible phosphorylation sites by the NetPhos 2.0 and NetPhosK servers. The NetPhos 2.0 server produces neural network predictions for serine, threonine, and tyrosine phosphorylation sites in eukaryotic proteins [44]. This analysis predicted that Tyr233 in CLIC1 has a phosphorylation potential of approximately 95% (Supplementary Figure 5). The NetPhosK analysis [45] detects kinase-specific phosphorylation sites and predicts Tyr233 to be a potential SRC kinase phosphorylation site with a moderate score of 53%. This result may have significance in view of the conservation of Tyr233 in the CLIC fold, together with experimental observations that some CLICs interact with kinases and that CLIC5B interacts with c-src kinase [9].

### 3.4. Binding of Glutathione

**Figure 5:**
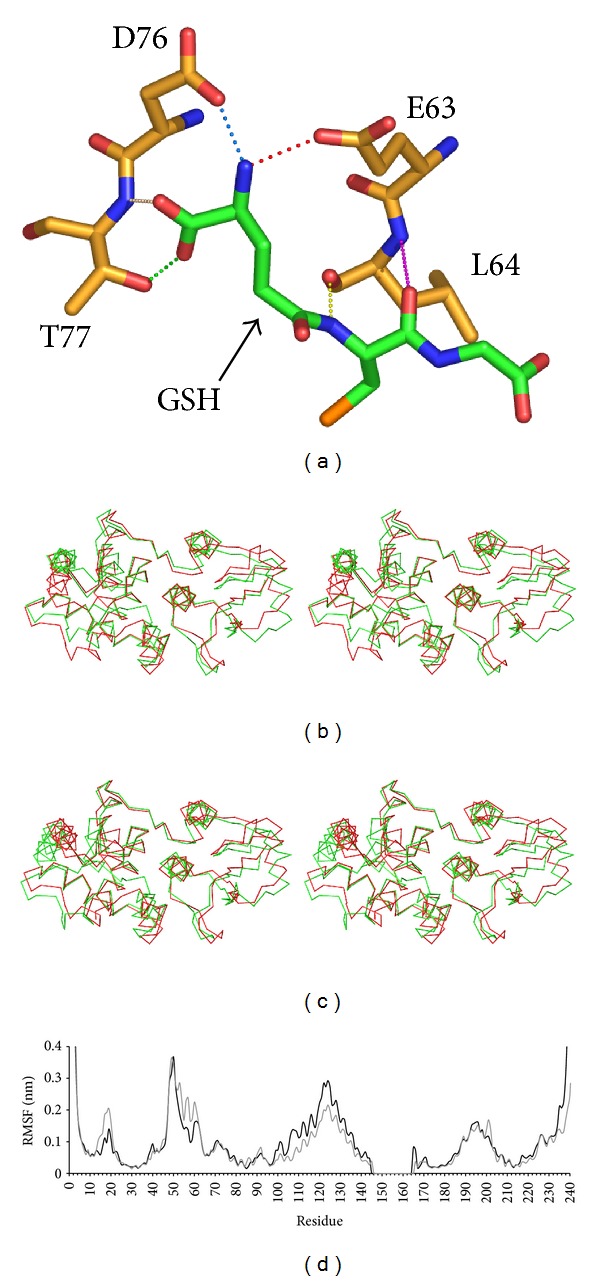
Binding of GSH and mechanism of release from the noncovalently bound state. (a) Interactions of GSH with CLIC1. Final frame from the cov1 simulation (*t* = 112.5 ns). GSH and protein regions which interact with the ligand are shown in stick representation with nitrogen blue, oxygen red, and sulphur orange; carbon atoms of GSH are green and those of CLIC1 are tan. Residues and ligand are as labeled. Interactions discussed in the text are indicated by dotted lines and coloured as in Supplementary Figure 6 (Supplementary Material available at http://dx.doi.org/10.1155/2013/170586). (b) EV2 and (c) EV3 from PCA of the combined apo1 and cov1 simulations. Stereo image of the C*α* trace of the superimposed maximum (green) and minimum (red) projection structures. The view is roughly orthogonal to that in Figure 1, equivalent to the perspective from the bottom of the page in Figure 1, along the axis of *α*-helix 1; the N-terminal subdomain is on the right and the C-terminal subdomain on the left. (d) Concerted motions in the ncv2 and ncv3 simulations show a peak in the most concerted 50% of C*α* atom fluctuations for residues 60–63. Per residue fluctuations due to EVs 1–5 from PCA analysis of the C*α* trajectory of simulation ncv2 (black) and EVs 1–4 from simulation ncv3 (grey). Residues 145–165 were not included in the PCA analysis.

The CLIC N-domain has a well-conserved glutaredoxin-like GSH binding site and in the crystal structure GSH is covalently attached to Cys24 via a disulfide bond to the conserved active site cysteine. The CLIC1 binding site has fewer interactions between GSH and the protein than observed in GSTs, with no interactions with either the terminal nitrogen of the *γ*-glutamyl moiety or the cystyl carbonyl group, which are present in all structures of GST-GSH complexes. However, in both of the present simulations of the covalently GSH-bound CLIC1, protein-ligand interactions analogous to those observed in GSTs are formed. Thus, the terminal nitrogen of the GSH *γ*-glutamyl moiety forms hydrogen bonds with the side chains of Asp76 and Glu63 (Figure 5(a)). These interactions are analogous to those observed in GSTs in that Asp76 and Glu63 are situated in equivalent positions in the shared conserved fold to an aspartate and glutamine residue, respectively, in GSTs.

It should be noted that the crystallised protein (1K0N) contains a glutamate at position 63, whereas the Swiss-Prot database entry for human CLIC1 (Accession no. O00299) contains a glutamine. This difference is due to a sequence error in the original CLIC1 clones resulting in Glu63. Although the residue at this position varies among CLICs, it is nearly always glutamine, asparagine, or histidine. Nonetheless, the simulations show that a glutamate side chain is able to form a stable salt bridge (Supplementary Figure 6) with the terminal nitrogen of the GSH *γ*-glutamyl moiety of the ligand, thus performing a similar function to the canonical residue at this position in GSTs. We have solved the crystal structure of the true wild-type sequence with Gln63 (Phang et al., unpublished results). The structure is essentially identical to the Glu63 mutant structure.

In addition to the interactions of Asp76 and Glu63 with the ligand, hydrogen bonds between the backbone amide of the GSH cystyl moiety and the carbonyl of Leu64, and between the carboxyl group of the *γ*-glutamyl moiety and the side chain of Thr77 (Figure 5(a)), observed in the crystal structure, remain stable throughout both covalently ligand-bound simulations (Supplementary Figure 6). The GSH cystyl carbonyl group interacts with the backbone amide of Leu64, also in a manner analogous to that observed in GSTs, although the interaction is more volatile than those involving the terminal nitrogen of the GSH *γ*-glutamyl moiety. In addition, a hydrogen bond forms intermittently between the carboxyl of the *γ*-glutamyl moiety and the backbone amide of Thr77. Notably, conserved Arg29, which corresponds to the conserved Lys/Arg in glutaredoxins [43] does not bind to the ligand as occurs in GSTs, consistent with distinct mechanisms with respect to their active sites between the two classes of proteins [19]. 

### 3.5. Relative Motions of the N- and C-terminal Subdomains Are Correlated with the Ligand-Bound State

 In order to compare the apo and covalently ligand-bound states, we performed PCA analysis on the combined trajectories of simulations apo1 and cov1, using the initial 75 M timesteps from each trajectory. PCA mode 1 from this analysis was essentially identical to principal mode 1 from PCA of the apo1 simulation alone, discussed above (subspace overlap >94%). The same result was also found when PCA of all trajectories combined was performed (subspace overlap >91%). This indicates that this mode describes the largest amplitude global motion that occurred in any of the simulations.

Modes 2 and 3 from PCA analysis of the combined trajectories of simulations apo1 and cov1 described two distinct motions, each involving a characteristic relative movement between the N- and C-terminal subdomains of the protein (Figures 5(b) and 5(c)). The first of these involved a global twisting along the long axis of the protein, which runs roughly along the groove between the N- and C-terminal domains. In the second motion, an opening and closing of the cleft that divides the N- and C-terminal domains occurs resulting in a breathing motion around the mouth of the active site. Supporting the idea that these motions may have a functional role, principal modes 1 and 2 from the anisotropic elastic network analysis (ANM), which shows direction of motion, show striking similarities with the twisting and breathing motions, respectively, observed in the MD simulations (Supplementary Movies 1–4).

PCA analysis of individual trajectories indicated that both the breathing and twisting motions occurred in all simulations. However, in comparing the apo and covalently ligand-bound states, the breathing motions are greater in the apo state while in the covalently ligand-bound state, the twisting motions are greater. This is shown by the fluctuations along EVs 2 and 3 for each trajectory (Supplementary Table 1). Although the trend is weaker for the second set of simulations, it is consistent both within trajectories and when comparing the apo and liganded states. Thus, the data suggest that the extent of each motion may be determined by the ligand-bound state.

### 3.6. Concerted Protein Dynamics and Ligand Release in the Noncovalently Bound State

To investigate the protein-ligand interaction, three simulations of the noncovalently ligand-bound state were performed. The first of these was started from the crystal structure, the second was started with the C-terminal residues modeled as a continuous *α*-helix, as described above, and the third started from the crystal structure with ligand-binding residue Glu63 changed to the wild-type glutamine. Significantly, in all three noncovalently bound GSH simulations, the ligand was completely released from the protein within 75 ns. In the ncv1 simulation, the hydrogen bond between the ligand and the side chain of Glu63, observed in the covalently bound simulations remained unformed, as in the crystal structure, and the bond between the ligand and the side chain of Asp76 was lost within 4 ns; the ligand completely disengaged within 10 ns.

In simulations ncv2 and ncv3, in which the ligand remained bound for approximately 70 ns and 20 ns, respectively, protein interactions with the ligand are the same as in the two covalently bound simulations, with the exception of the hydrogen bond between the backbone amide of the GSH cystyl and the carbonyl oxygen of Leu64 (Supplementary Figure 6). This latter interaction remains stable throughout both covalently ligand-bound simulations but forms only transiently in simulations ncv2 and ncv3; in the covalently bound state it appears to be stabilised by the covalent attachment of the ligand cystyl moiety to the protein (Figure 5(a)).

From the fact that in both simulations ncv2 and ncv3 the ligand had the opportunity to form the same complement of interactions with the protein as observed in the covalently attached ligand, we conclude that the disengagement of the ligand was not due to artefacts relating to the formation of required protein-ligand interactions, either during or following the equilibration. In addition, we note that ligand release occurred in all noncovalently bound simulations, regardless of whether minimal counterions were employed as in ncv2 or a 0.2 M NaCl solution was used as in ncv1 and ncv3.

Visual inspection of the simulation trajectories suggested that the mechanism of ligand release might involve dynamics of the loop joining *α*-helix 2 and *β*-strand 3 (residues 59 to 65), which affects the interactions of residues Glu63 and Leu64 with the ligand. Motions of this loop are allied to high fluctuations of the *α*-helix 2 region (residues 50–60; Figure 1(b)). PCA of the trajectories of the ncv2 and ncv3 simulations shows a peak for residues 60–63 in the per residue plot of the most concerted 50% of C*α* atom fluctuations (Figure 5(d)), indicating that motions of residues 60–63 are part of concerted global dynamics of the protein.

Examination of the PCA of the ncv2 and ncv3 simulation trajectories reveals that the fluctuations of residues 60–63 are decomposed into a complex set of motions that alter their position relative to the ligand and to other residues comprising the ligand binding site. These motions would alter the geometry of the ligand-binding site and in doing so, possibly contribute to ligand release. In many instances, PCA modes 1–5 of the ncv2 and ncv3 trajectories involved alterations in the backbone dihedral *ψ* angle of ligand-binding residue residue Glu/Gln63, and ligand release was approximately coincident with sustained changes in this angle in both the ncv2 and ncv3 simulations (Supplementary Figures 7(a) and 7(b)).

We performed PCA of the combined ncv2 and ncv3 simulation trajectories, not including frames following the onset of ligand release (*t* = 67 ns and *t* = 19.85 ns for ncv2 and ncv3, resp.). EV4 from this analysis, which accounted for more than 10% of all C*α* atom fluctuations, included alterations in the backbone dihedral *ψ* angle of ligand-binding residue Glu/Gln63. This mode also showed 12.7% and 52.3% subspace overlap with EVs 2 and 3, respectively, from the PCA of the combined apo1 and cov1 simulations (see above), which both include significant motions of residues 60–63 (Figures 5(b) and 5(c)). The projection of EV4 from PCA of the combined ncv2 and ncv3 simulation trajectories on the complete ncv2 and ncv3 simulation trajectories revealed that ligand release was coincident with the maximum projection(s) in both cases (Supplementary Figures 6(c) and 6(d)). Thus, a significant global concerted motion that was detected prior to ligand release reached its fullest extent coincident with ligand release, in both the ncv2 and ncv3 simulations.

### 3.7. The Foot Loop is Conformationally and Dynamically Variable

**Figure 6:**
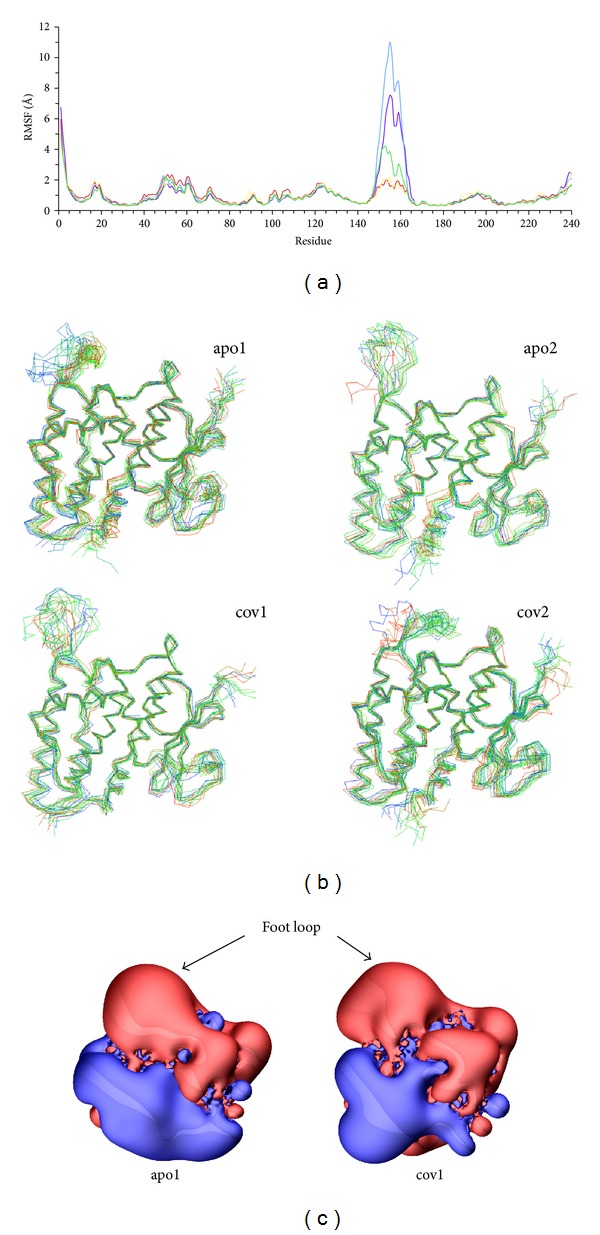
Structure and dynamics of the foot loop. (a) Fluctuations of the foot loop. Per residue fluctuations of C*α* atoms during the simulations: apo1, red; apo2, blue; cov1, green; cov2, yellow; ncv2, purple. Fluctuations taken over the final 20 ns of each simulation relative to the average structure over the same period for the respective simulation. (b) Conformational variability of the foot loop. Superimposed trajectory frames taken at 7.5 ns intervals, coloured according to the time: start (blue); end (red). (c) Electronegativity of the foot loop. Electrostatic potential around the structure of CLIC1 is depicted as a surface representing isovalues; potential of +1 is coloured blue and −1 red. The view is similar to that in Figure 1. Final frame from each simulation was analysed.

 Analysis of the simulation trajectories shows that the foot loop (residues 147–164) undergoes the highest fluctuations within the protein (Figures 1(b) and 6(a)), consistent with the disordered nature of this region in the crystal structure. The foot loop was observed to be highly mobile and conformationally variable. This is depicted in Figure 6(b), which shows the C*α* trace at regular intervals over the course of the apo and covalently bound simulations. In simulations apo1 and cov2, the foot loop equilibrates to a generally more compact conformation than in simulations apo2 and cov1; in the latter simulations, it adopts a more extended and mobile conformation. The compact conformations observed in simulations apo1 and cov2 are not similar in terms of their secondary structure (Supplementary Figure 8), and although the lower mobility of this region in simulations apo1 and cov2 appears, at least in part, to be due to electrostatic interactions with the residues within the structural core of the protein, these interactions are not the same in the two simulations. The foot loop is notable also for the high negative electrostatic potential surrounding it (Figure 6(c)) and that it contains regions of high sequence variability among CLICs (Supplementary Figure 9).

## 4. Discussion

In order to investigate the role and molecular mechanism of CLIC1, we performed MD simulations of the three possible ligand-bound states of the molecule, using the crystal structure of GSH-bound human CLIC1 as the starting structure. For comparison with the results of the atomistic simulations, elastic network analysis of the CLIC1 structure was also performed. Two >100 ns MD simulations of the nonliganded state revealed significant alterations of the protein around and within the active site that resulted in direct interaction between conserved residue Tyr233, situated within the C-terminal helix 9, and the active site Cys24 thiol. These changes involved characteristic alterations in the orientation of the C-terminal *α*-helix and its extension at its C-terminus, and concomitant changes in *α*-helix 6 and the downstream loop encompassing residues 195–198. In two equivalent >100 ns simulations of the covalently glutathione-bound state, movement of Tyr233 toward Cys24 did not occur, nor did the other changes described in the apo simulation; this was also true when the C-terminal residues were modeled in an *α*-helical conformation in the starting structure. PCA of the combined trajectories of all simulations showed that the global change that occurred in the apo1 simulation was the largest amplitude concerted motion that occurred in all simulations. Moreover, elastic network analysis of the protein structure indicated that the conformational changes observed in the apo simulations involve motions intrinsic to the gross architecture and are thus likely to be functional.

Since the structures of both ligand-bound (1KON) and apo (1KOM) human CLIC1 are essentially identical (RMSD 0.5 Å; 6), the crystallographic analysis indicates that the starting structure used in the simulations is the lowest free energy conformation for both states. This appears to conflict with the findings of the simulations, in which the unliganded protein equilibrates to a distinct conformation. Nevertheless, crystallographic analysis provides evidence that the altered structure observed in the C-terminal residues in the equilibrated apo1 simulation, which appears to be part of the overall conformational change, could represent a physiological state.

Several CLIC protein crystal structures indicate that the C-terminal region after helix 9 is less ordered than the remainder of the protein. The C-terminal region (234–241) was unresolved in a structure of oxidised CLIC1 (1RK4 [46]), whereas in two structures of human CLIC3 (3FY7, 3KJY [47]), the conserved C-terminal tyrosine (equivalent to Tyr233 in CLIC1) is the penultimate resolved residue, with the C-terminal tail lacking ordered structure. In addition, in three structures of human CLIC2A, temperature factors increase for residues following *α*-helix 9 and for CLIC4; some structures also show increased B-factors after helix 9. These data are consistent with the existence of alternative conformations for the C-terminal residues.

Additionally, recent EPR experiments show that the CLIC1 C-terminal region switches from an ordered to a disordered state when the protein interacts with lipid membranes [48]. In these experiments, a spin label has been attached to the conserved Cys223 between helices 8 and 9 in the oxidised CLIC1 dimer state. In this state, the spin label shows low mobility, consistent with a structured region of the protein. On the addition of liposomes, the spin label becomes completely mobile. In the apo1 simulation, changes in the region between helices 8 and 9 are important to the reorientation of the C-terminal helix 9, and fluctuations in this region are significantly lower in the cov1 simulation (Figure 6(b)). Thus, the EPR data support the observed altered mobility of this region observed between different states in the simulations and the possibility that alterations in the orientation of the C-terminal helix 9 occur and may have a physiological role.

The results of simulations may be interpreted as indicating that physiologically, the structure observed in the crystal is in equilibrium with an alternative state, where the C-terminal *α*-helix forms to the very terminus. If this forms when Cys24 is covalently bound to glutathione, then there is nothing to stabilise the extended helix state. However, in an apo structure, Tyr233 can hydrogen bond to Cys24 and hence add stability to the extended helix form.

What pins down the C-terminal extended structure in the CLIC proteins? In CLIC1, 4, 5 and 6, two conserved non-polar residues, Ala237 and Leu240 (Met in CLIC6), sit in hydrophobic pockets formed by helices 4, 5, 6, and 9. In CLIC2, Leu240 is replaced by Gln245, which packs in the same pocket, which is now polar, with side chain hydrogen bonds to Asn132. These structures are not present in CLIC3 and the extended C-terminal structure beyond the conserved tyrosine in helix 9 is not observed.

The release of the extended C-terminal structure in the CLIC1 MD simulations is correlated with structural changes in the hydrophobic pockets to which Leu240 and Ala237 bind. Concomitant to the release of the C-terminus, significant changes are observed in the loop joining helix 6 to helix 7 (residues 195–198), which forms part of the pockets. This region directly follows the putative nuclear localization sequence Lys192-Lys193-Tyr194-Arg195, which needs to adopt a linear conformation in order to bind importin *α* for nuclear transport [49]. Thus, the structural changes that result in the release of the C-terminal extended region may be prerequisite for the exposure of the putative nuclear localization sequence and hence nuclear transport of the CLIC protein.

Calculation of the pKa of the active site cysteine thiol indicated that is less reactive in the conformation in the >100 ns equilibrated apo structure than in the conformation observed in the starting structure. Thus, the proximity between Tyr233 and Cys24, observed in the apo simulations, appears to induce and/or stabilise a less reactive conformer of the active site thiol. Residue Tyr233 is conserved in all vertebrate CLIC sequences to date. Its ability to contact the active site cysteine in a ligand-dependent manner, as indicated by the simulations, suggests that it may have a catalytic role, possibly in breaking and/or formation of the covalent bond between Cys24 and the GSH ligand, and/or the transfer of GSH to or from another molecule. The possible importance of Tyr233 in reactions of the active site is also supported by the NetPhos neural network analysis, which suggests that it may be a tyrosine kinase phosphorylation site. The less reactive conformation of the active site cysteine, together with the observation that the side chain of Phe26 transiently occupies the ligand-binding space in the apo simulations, suggests that the probability of spontaneous ligand binding is reduced in the unliganded state.

The simulation results indicate that interactions of GSH with the protein in the covalently bound state are similar to those observed in GSTs. Notwithstanding the existence of an intact glutaredoxin-like GSH binding site, in three simulations of the noncovalently bound state, the ligand is rapidly released from the protein. This appears to occur by a mechanism involving concerted global dynamics. The rapid release of GSH in all three noncovalently ligand-bound simulations is consistent with experimental data which showed that non-covalent binding of GSH to CLIC1 is weak (*k*_*d*_ > 10 mM; 6) compared to that observed in GSTs, which show strong GSH binding (*k*_*d*_~*μ*M [50]). Together, the data suggest that, when the ligand is absent or present but not covalently bound, CLIC1 is in a state conformationally and dynamically that is not conducive to spontaneous covalent binding of GSH to the ligand binding site.

PCA analysis of the simulation trajectories detects and distinguishes two different kinds of motions between the N- and C-terminal subdomains. In the nonliganded state, an opening and closing of the gap around the active site formed by these subdomains are the predominant motion, whereas in covalently ligand bound state, a twisting motion with respect to these subdomains along the long axis of the protein is the predominant motion. Significantly, EN analysis modes 1 and 2 show remarkable similarities with the twisting and breathing motions between the N- and C-terminal subdomains, respectively, indicating that they are fundamental to the architecture of CLIC1 and are thus likely to be of functional significance.

Since GSH is unlikely to bind covalently to CLIC spontaneously under physiological conditions, the conclusion follows that covalent binding of GSH to CLIC requires interaction with another protein. Such an interaction could alter the structure and/or dynamics of CLIC to increase the binding affinity for GSH and/or to transfer the ligand. The region of the mouth around the active site and the associated cleft between the N- and C-terminal domains, which undergoes the greatest changes in the simulations, is expected to be the site of docking of other molecules. Thus, it seems reasonable to suggest that the breathing motion in the unliganded state could aid the binding of a glutathione transferase, while the twisting motion in the covalently ligand-bound state would induce its release. 

The simulations also show that the foot loop region of the protein (residues 147–164) is able to adopt both extended, highly mobile conformations, as well as more compact, less dynamic conformations. The high conformational variability of the foot loop appears similar to that described for intrinsically disordered or unstructured regions of other proteins. In many cases, natively unstructured regions are involved in recognition and binding to other proteins or macromolecules; a relatively unstructured region can have a greater capture radius for a specific binding site than the folded state with its restricted conformational freedom [51]. Unstructured regions also have the potential to bind multiple targets. Indeed, the behaviour of the foot loop in simulations apo2 and cov1 resembles broadly the description of the process of recognition and binding of unstructured regions to their targets as a “fly casting” mechanism [52]. The high electronegativity of the foot loop (Figure 6(c)) could aid in the weak longer-range nonspecific binding, proposed to be the initial phase of the fly casting binding process [52]. In this context, it is interesting that the secondary structure that forms in the foot loop is more fragmented temporally in the simulations with 0.2 M NaCl than in the second set of simulations with minimal counterions (Supplementary Figure 8). This suggests the possibility that external electrostatic interactions may influence the structure of the region. Since the foot loop contains regions of high sequence variability among CLICs (Supplementary Figure 8), it seems possible that it may be part of a recognition interface with other proteins. However, since the behaviour of the foot loop appears uncorrelated with the ligand-bound state, what role this might play is uncertain.

The data do not appear to shed any light on the way in which CLIC might undergo a radical conformational change and form a transmembrane chloride channel, with the rms deviation of the C*α* atoms remaining <3 Å for each simulation. This may be due to the limited length of the simulations. Alternatively, CLIC1 may require interaction with another protein and/or the membrane to refold and insert into the membrane.

## Supplementary Material

Suppl. Table 1 details contributions to the ionisation energy of residue C24 at pH7. There are an additional eight Supplementary Figures, most of which have several panels. These figures describe: the time course of conformational changes during the simulations; the rotation of crucial residues; the prediction of phosphorylation sites; protein-ligand interactions; conformational changes that accompany ligand release; secondary structure within the foot loop; and sequence variability within CLIC proteins. The table and figures have explanatory legends and they are discussed throughout the paper.

## Abbreviations

CLIC: Chloride intracellular channel
MD: Molecular dynamics
RMS: Root mean square
PCA: Principal component analysis
EV: Eigenvector.
