# Supplementary material for: Computational Analysis of the Soluble Form of the Intracellular Chloride Ion Channel Protein CLIC1
Source: Biomed Res Int. 2013 Sep 8;2013:170586. doi: 10.1155/2013/170586 (PMC3780514; doi:10.1155/2013/170586)
Supplement: Supplementary file 1 — Suppl. Table 1 details contributions to the ionisation energy of residue C24 at pH7. There are an additional eight Supplementary Figures, most of which have several panels. These figures describe: the time course of conformational changes during the simulations; the rotation of crucial residues; the prediction of phosphorylation sites; protein-ligand interactions; conformational changes that accompany ligand release; secondary structure within the foot loop; and sequence variability within CLIC proteins. The table and figures have explanatory legends and they are discussed throughout the paper. [file 170586.f1.zip › 170586.f1/clic_supplfigs_amg_2738_563296.pdf]

SUPPL. FIGURE 1

A

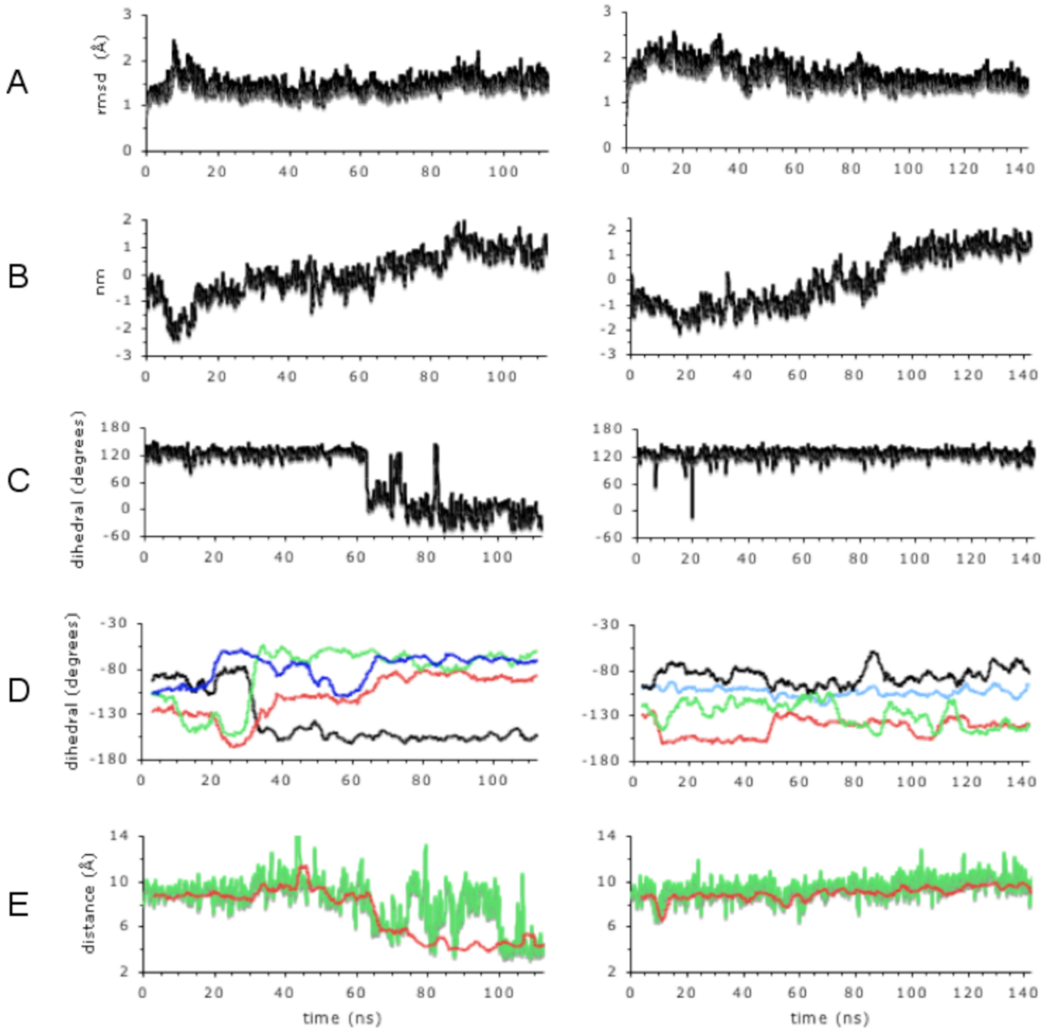

Time course of conformational changes in the second apo and second covalently ligand-bound simulations in which the C-terminal residues 226-241 were modelled in an α-helical conformation in the starting structure. Data from the unliganded simulations are on the left and those from the covalently ligand-bound protein are on the right. All measurements taken at 150ps intervals. (A) rms deviation of Cα atoms from the starting structure (not including foot loop residues 146-165 or residues 1-5; (B) projection of eigenvector 1 from the Cα trajectory (not including foot loop residues 146-165); (C) backbone ϕ dihedral angle of residue N23; (D) backbone ψ dihedral angles of residues 194 (blue), 195 (red), 197 (black) and 198 (green); (E) distance between the hydroxyl oxygen of Y233 and the sulphur atom of C24 (green) and moving average over 20 150ps intervals of distance between the hydroxyl oxygen of Y233 and the sidechain β-carbon atom of C24 (red).

B

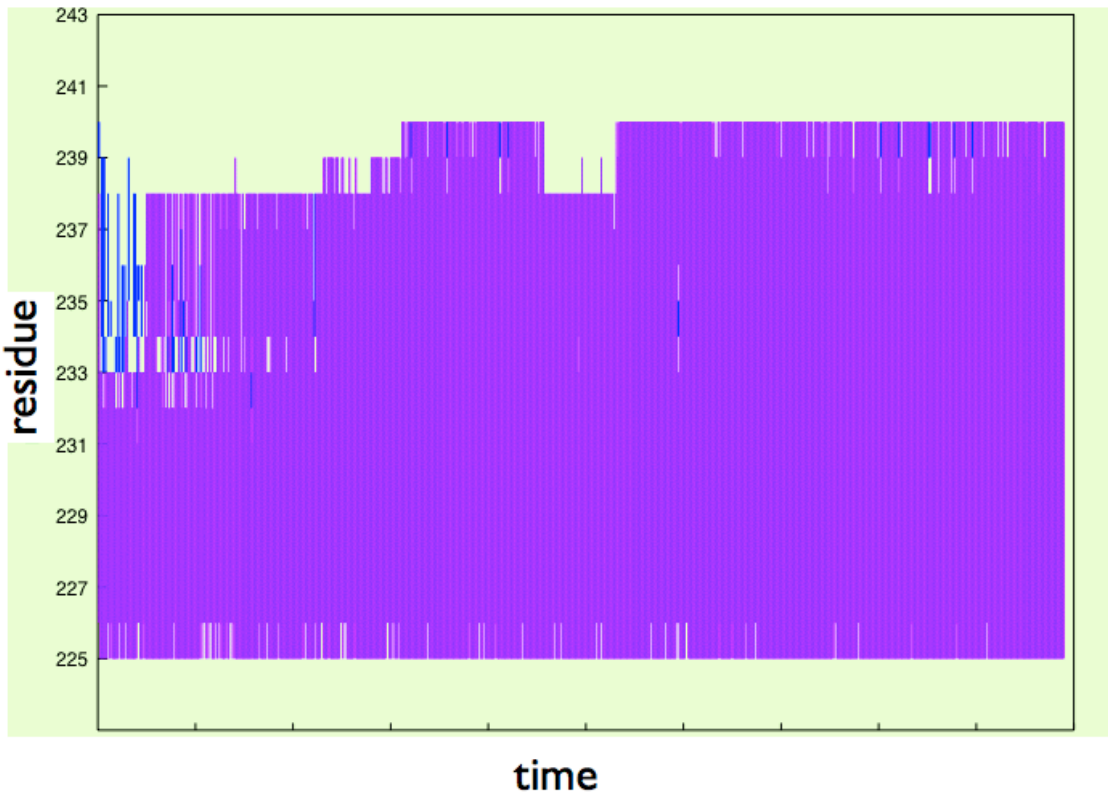

Secondary structure within the C-terminal residues in simulation apo2.

Time course of secondary structure within the segment comprising residues 225-241 in the apo2 simulation. 1-4 α-helix (purple) and 1-3 helix (blue).

SUPPL. FIGURE 2

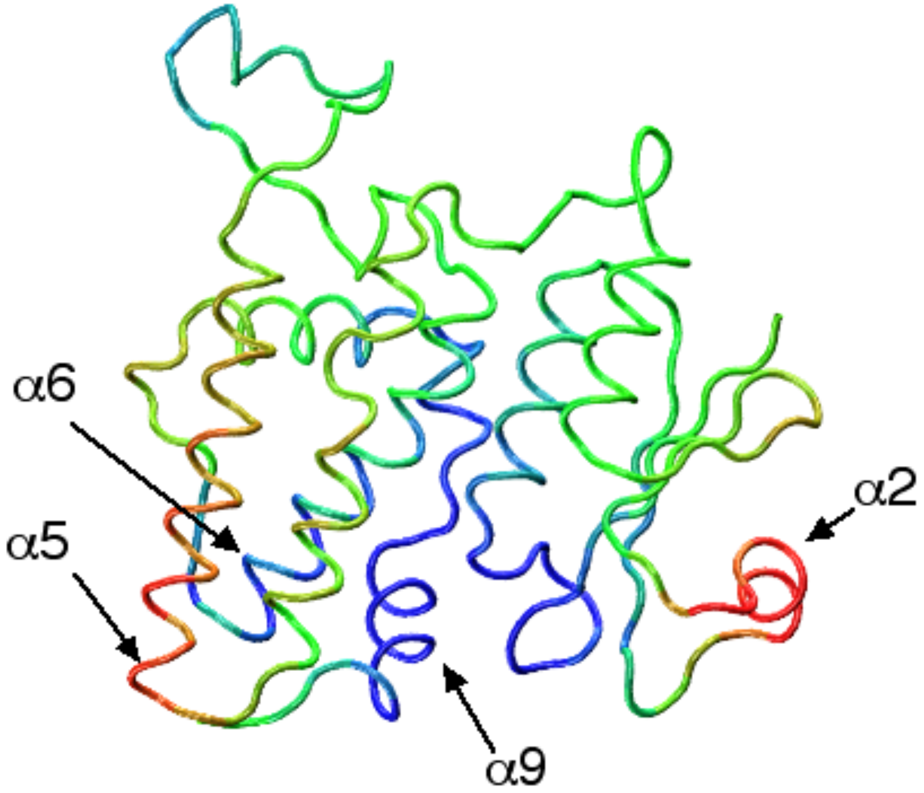

GNM principal mode 5. Tube representation of CLIC1 coloured according to the components of eigenvector 5 from the GNM analysis: blue, negative; red positive. Residues with the same colour undergo correlated motions (same direction) while blue and red residues undergo anticorrelated (opposite direction) motions. View is similar to that in Figure 1.

SUPPL. FIGURE 3

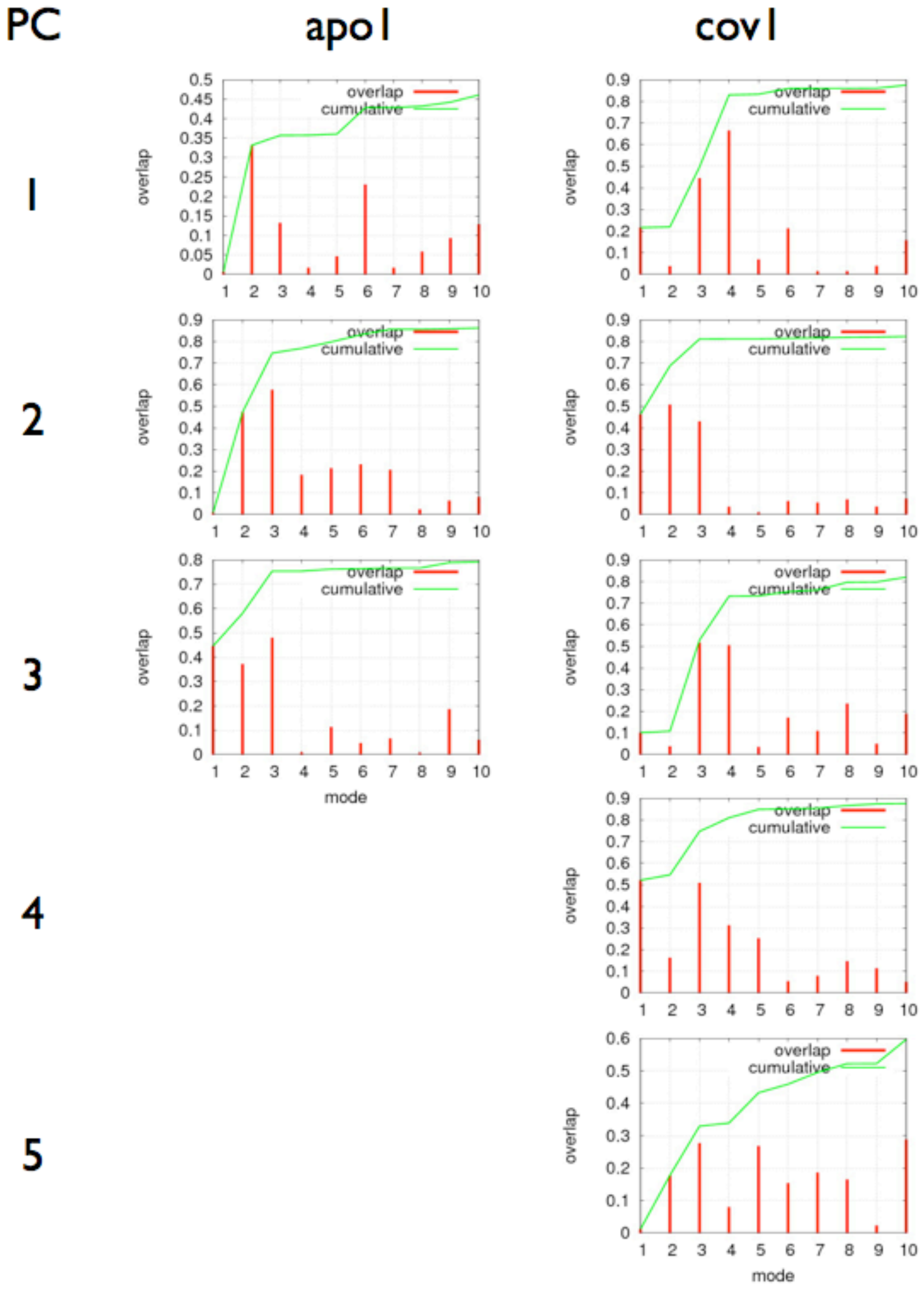

GNM principal modes contribute dominantly to the most concerted fluctuations in the simulations. The largest amplitude conformational changes during the apo1 and cov1 simulations, as described by PCA of Cα atom fluctuations, were analysed for the contribution of GNM modes to the motion described by the PCA mode. Each plot shows the overlap of GNM modes calculated from the minimum projection structure to the transition to the maximum projection structure, for PCA modes 1-3 from the apo1 simulation, and PCA modes 1-5 from the cov1 simulation; in each case these sets of modes encompass the most concerted 50% of Cα atom fluctuations in the simulation. For all modes except PC mode 5 from the cov1 simulation, one of the first 5 GNM modes shows the highest overlap of all GNM modes with the PC mode. In all cases except for PC mode 1 from the apo1 simulation and PC mode 5 from the cov1 simulation, the first 5 GNM modes contribute to 75% or more of the motion. GNM modes are numbered 1-10 in the plots and shown as red bars with the cumulative contribution of each GNM mode to the PC mode shown as a green line. PCA modes from the simulations are numbered 1-5 down the left of the figure.

SUPPL. FIGURE 4

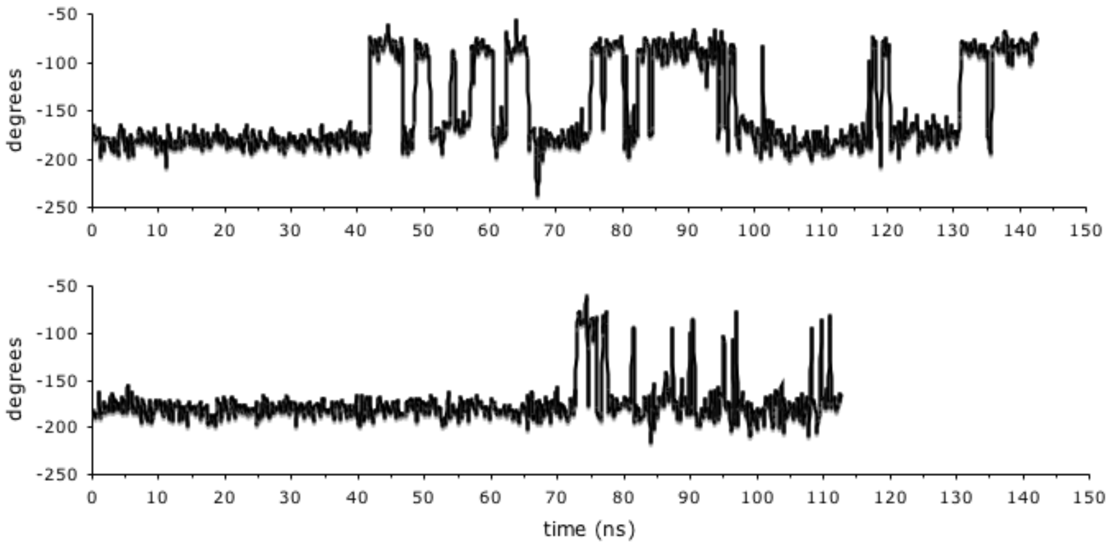

Rotation of F26 sidechain into the GSH binding site.
Time series of the dihedral angle between residue F26 atoms C, Cα, Cβ and Cγ. Dihedral angles in the range -50 to -100 indicate the conformation in which the phenylalanine sidechain is rotated into the region occupied by the covalent bond to the ligand when covalently bound (see Fig. 3 bottom right). Top, apo1 simulation; bottom, apo2 simulation.

SUPPL. FIGURE 5

Predicted phosphorylation sites in CLIC1.

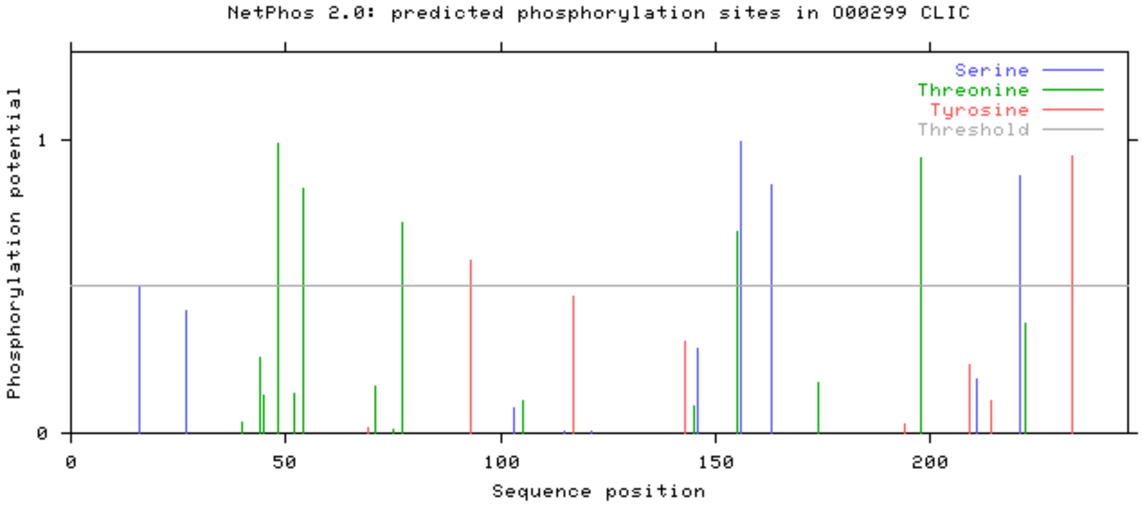

Output from the NetPhos 2.0 neural network algorithm for SwissProt accession code O00299 human CLIC1, showing per residue predicted probabilities for serine, threonine and tyrosine phosphorylation sites. Y233 is rightmost red line.

SUPPL. FIGURE 6

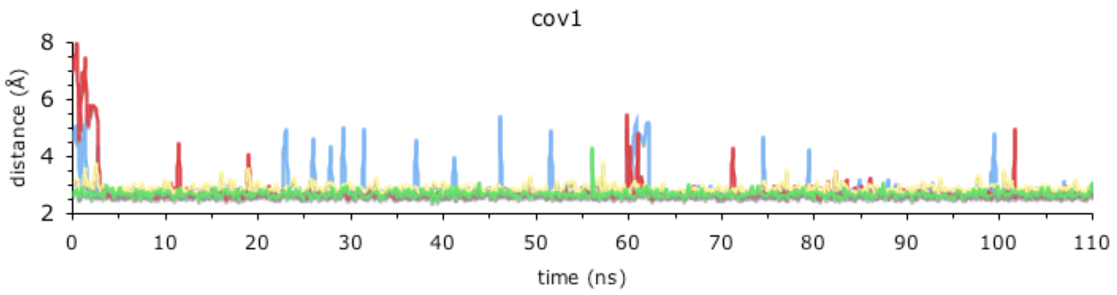

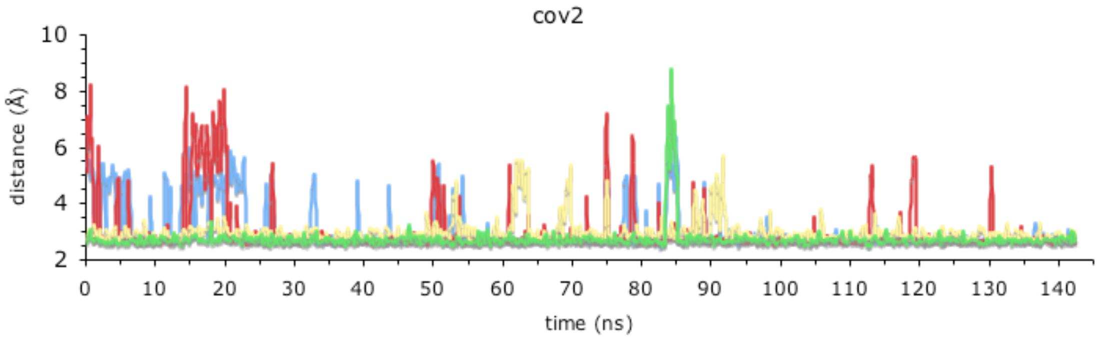

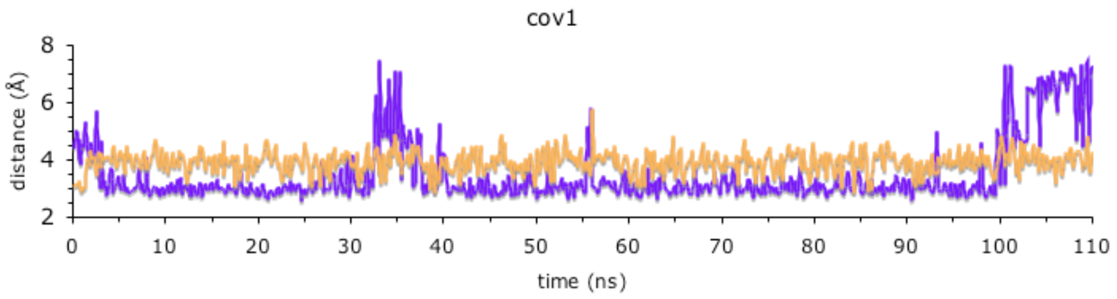

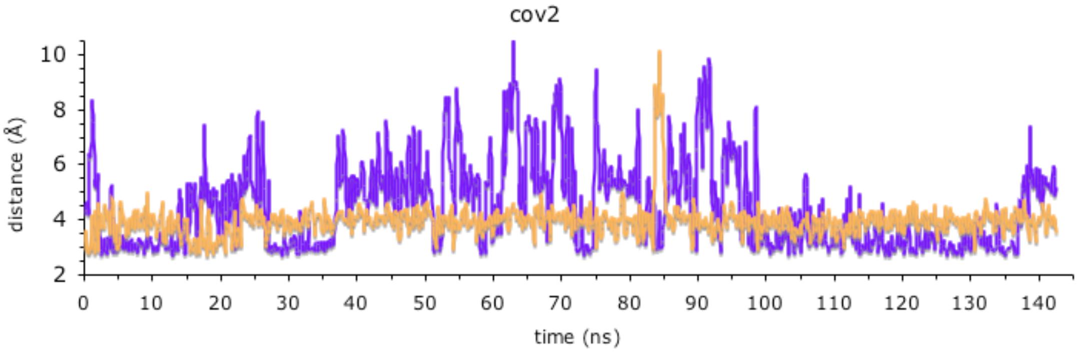
cov2
distance (Å)
time (ns)

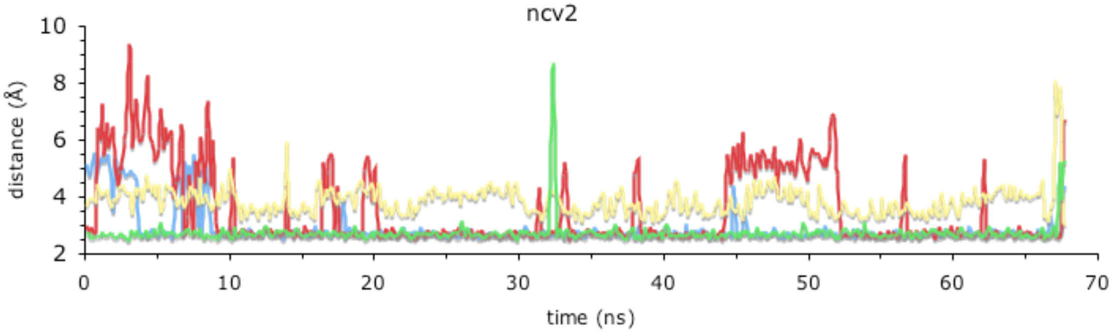
ncv2
distance (Å)
time (ns)

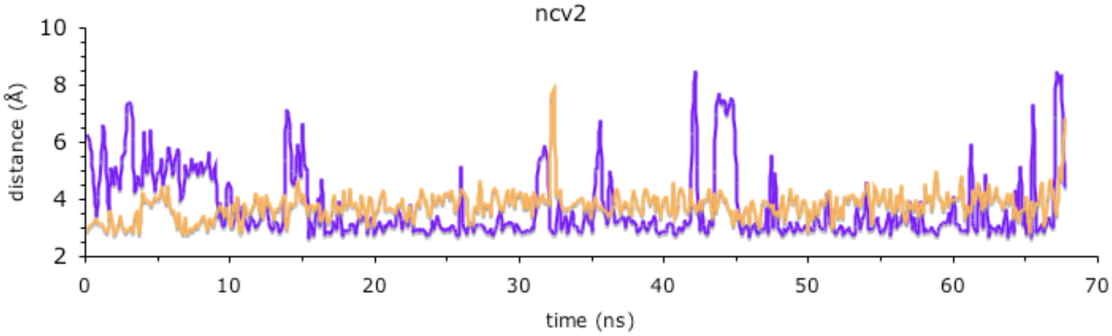
ncv2
distance (Å)
time (ns)

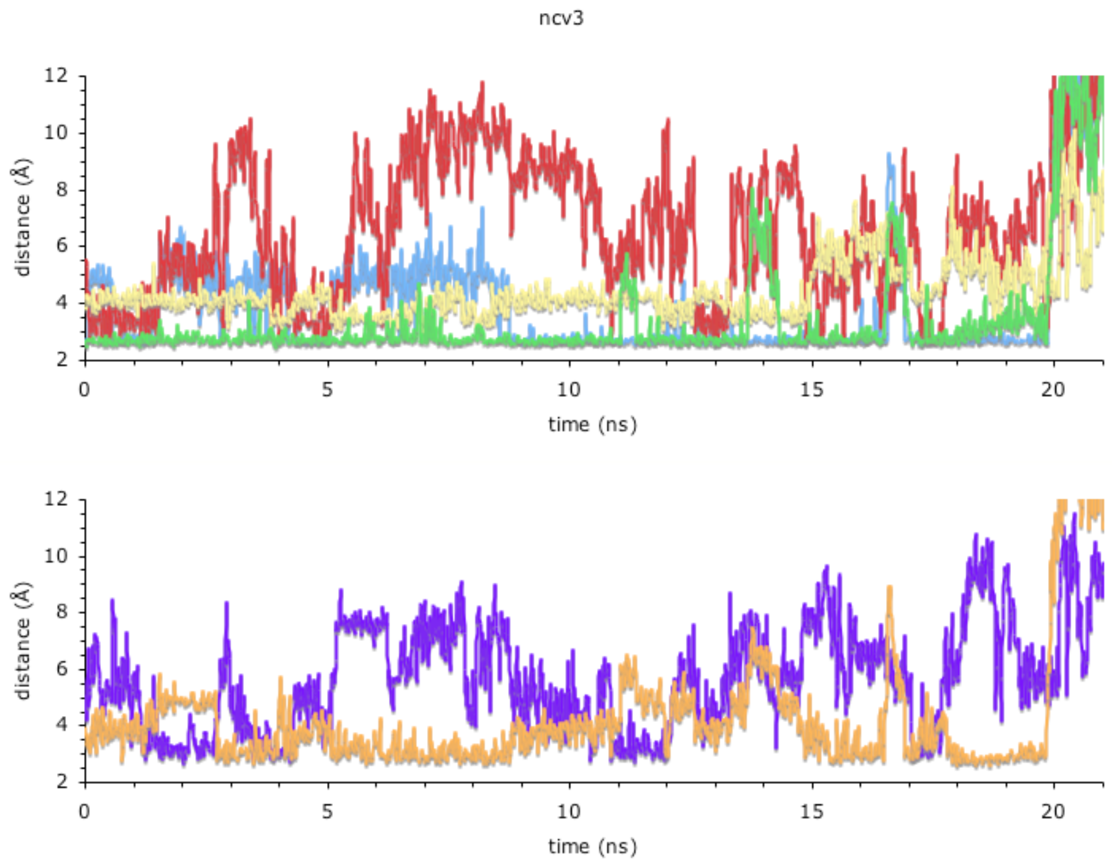

Protein-ligand interactions in the ligand-bound simulations. Time series of distances between atoms of GSH and the protein; D76 sidechain oxygen and γ-glutamyl nitrogen (blue), E63 sidechain oxygen and γ-glutamyl nitrogen (red), L64 backbone oxygen and cystyl nitrogen (yellow), T77 sidechain oxygen and γ-glutamyl carboxyl oxygen (green), L64 backbone nitrogen and cystyl oxygen (purple), T77 backbone nitrogen and γ-glutamyl carboxyl oxygen (tan). For D76 and E63 the shorter of the two distances to the sidechain oxygens is given. Data for each simulation is presented in pairs: top pair, covalently ligand-bound simulation 1; middle pair, covalently ligand-bound simulation 2; third pair, non covalently ligand-bound simulation 2; fourth pair, non-covalent ligand-bound simulation 3. Data taken at 150 ps intervals.

SUPPL. FIGURE 7

A

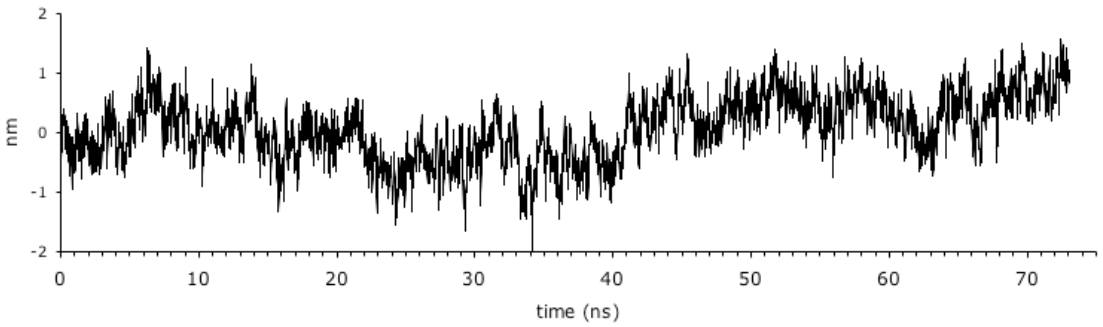

B

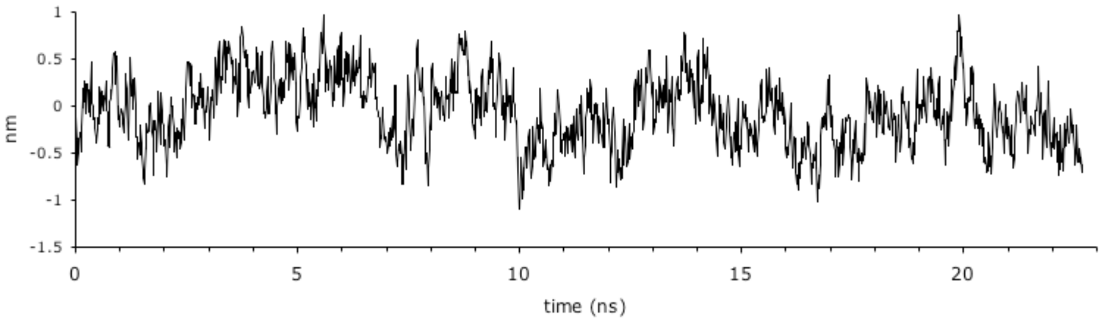

C

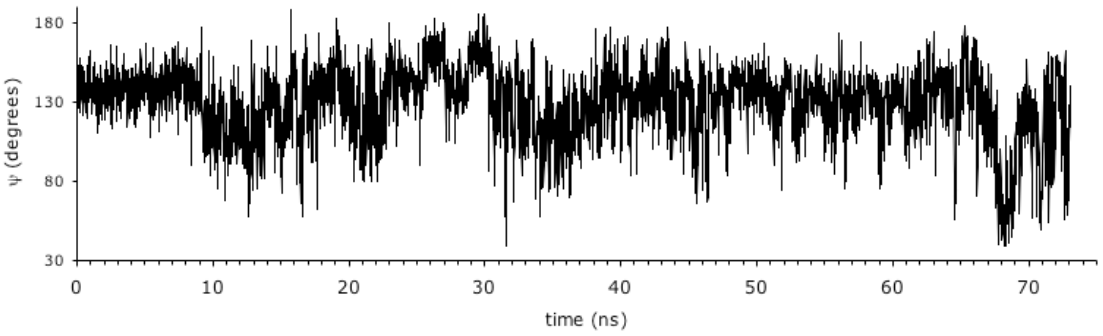

D

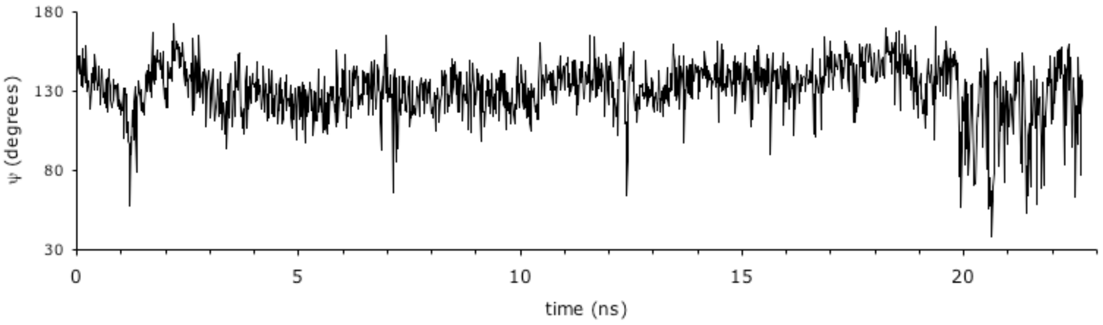

Protein conformational changes and ligand release in simulations ncv2 and ncv3.

(A) Time series of backbone dihedral ψ angle of ligand-binding residue Glu63 in simulation ncv2; (B) Time series of backbone dihedral ψ angle of ligand-binding residue Gln63 in simulation ncv3: (C) Time series of projection of EV4 from PCA of the combined ncv2 and ncv3 simulation trajectories on the trajectory of simulation ncv2; (D) Time series of projection of EV4 from PCA of the combined ncv2 and ncv3 simulation trajectories on the trajectory of simulation ncv3.

SUPPL. FIGURE 8

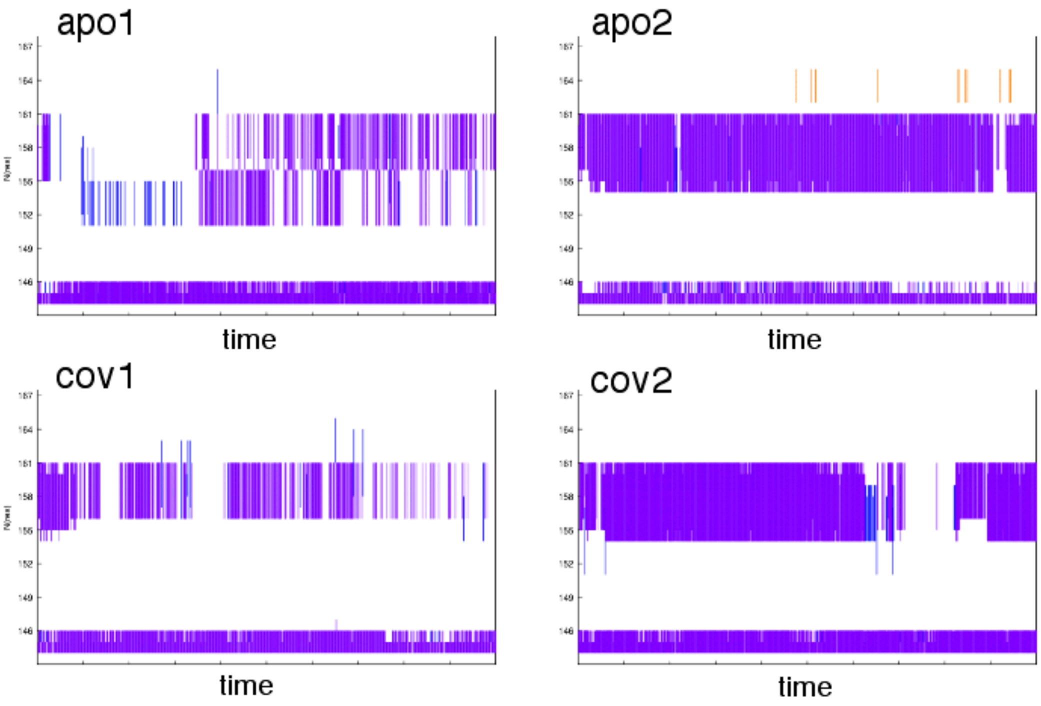

Secondary structure within the foot loop (residues 145-165).
Time course of secondary structure within the segment comprising residues 145-165 in the apo and covalently ligand-bound simulations. 1-4 α-helix (purple), 1-3 helix (blue), antiparallel β-sheet (tan).

SUPPL. FIGURE 9

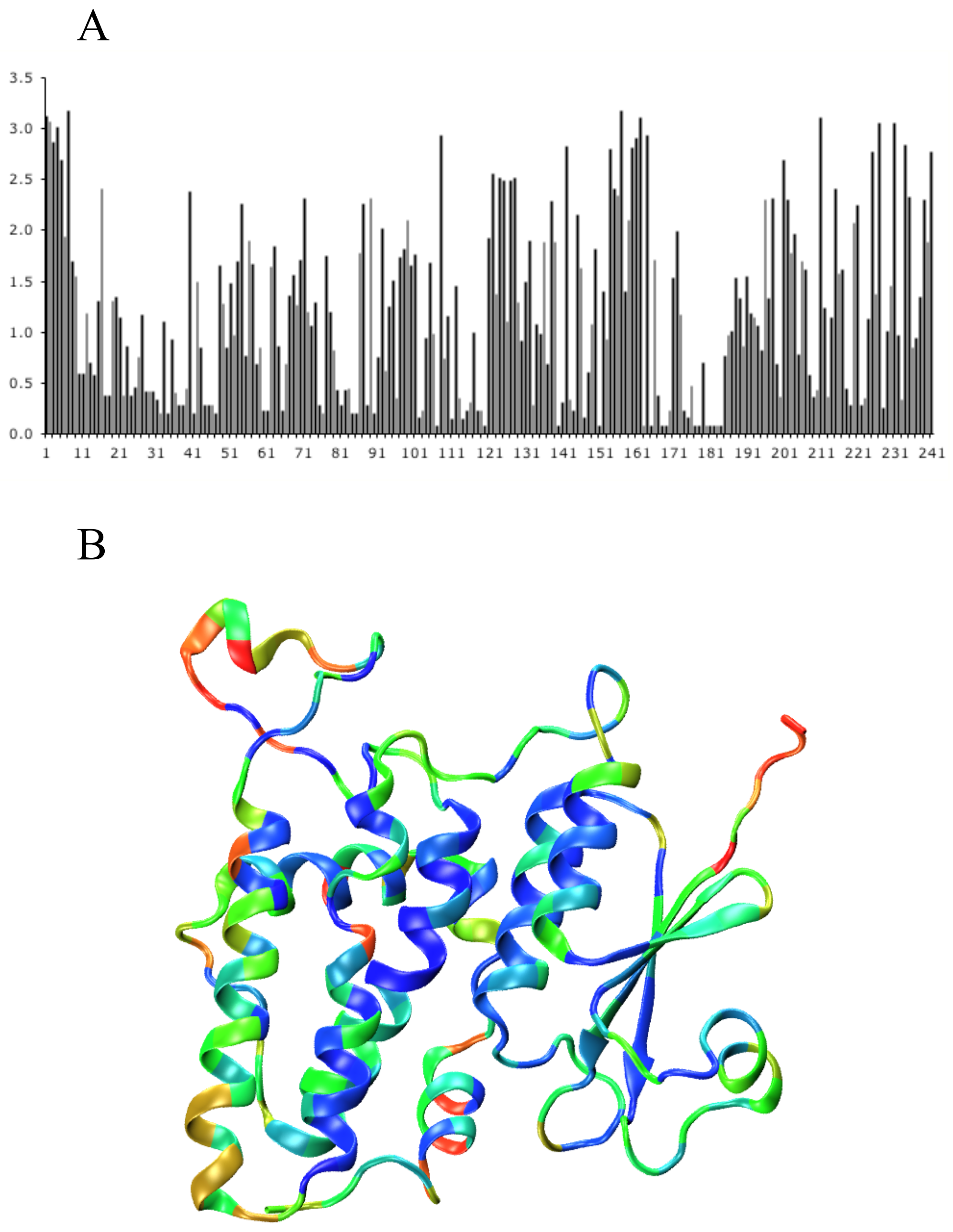

Sequence variability within CLICs. Sequence variabilty at each position is expressed as the Shannon entropy (H), measured over the closest 100 sequences to human CLIC1 as determined from a BLAST search of the SwissProt protein sequence database. H values range from 0 (only one residue in present at that position) to 4.3 (all 20 residues are equally represented in that position). (A) Plot of per residue values of Shannon entropy; (B) Values mapped to the structure of CLIC1, colour coded according to the Shannon entropy, with low values blue and high red. The view is similar to that in Figure 1.
