# Supplementary material for: Computational Analysis of the Soluble Form of the Intracellular Chloride Ion Channel Protein CLIC1
Source: Biomed Res Int. 2013 Sep 8;2013:170586. doi: 10.1155/2013/170586 (PMC3780514; doi:10.1155/2013/170586)
Supplement: Supplementary file 1 — Suppl. Table 1 details contributions to the ionisation energy of residue C24 at pH7. There are an additional eight Supplementary Figures, most of which have several panels. These figures describe: the time course of conformational changes during the simulations; the rotation of crucial residues; the prediction of phosphorylation sites; protein-ligand interactions; conformational changes that accompany ligand release; secondary structure within the foot loop; and sequence variability within CLIC proteins. The table and figures have explanatory legends and they are discussed throughout the paper. [file 170586.f1.zip › 170586.f1/CLIC_Suppl_Tble_1.pdf]

Suppl. Table 1. Contributions to the ionization energy of residue C24 at pH 7.

Terms	t=10ns	t=142.5ns
ebkb	-6.18	-2.62
dsol	4.96	1.84
pH&pK0	2.86	2.86
-TS	0.21	0.63
residues	-1.64	-0.69
TOTAL	0.26	2.06
LYS 13	-0.63	-0.67
ASP 17		0.22
SER 27	-1.52	
GLN 28	0.21	
ARG 29	-0.50	-0.24
GLU 63	0.27	0.21
ASP 76	0.26	
LYS 183	-0.35	-0.35
TYR 233		-0.43
GLU 228	0.32	0.26

Breakdown of energy terms that influence the free energy of ionization of the residue C24 thiol at pH 7. Values are in kcal/M, only terms greater than 0.2 kcal/M are shown. Negative values favour ionization. "ebkb" is the electrostatic interaction of the side chain to the backbone. "dsolv" is the loss of solvation energy. "pH&pK0" is the pH effect, which equals solution pH minus solution pKa of the residue if the residue is an acid. "-TS" is entropy effect. "residues" refers to the mean field interaction with residues at this pH. The bottom section of the table gives the breakdown of the residue contributions. Columns of values refer to analysis of two frames from the apo1 simulation at the indicated time points.
